# Development of a Sensory Flavor Lexicon for Mushrooms and Subsequent Characterization of Fresh and Dried Mushrooms

**DOI:** 10.3390/foods9080980

**Published:** 2020-07-23

**Authors:** SoonSil Chun, Edgar Chambers, Injun Han

**Affiliations:** 1 Department of Food and Nutrition, Sunchon National University, Jeonnam 57922, Korea; css@sunchon.ac.kr; 2 Center for Sensory Analysis and Consumer Behavior, Kansas State University, Manhattan, KS 66502, USA; 3 Division of Natural Product Research, Korea Prime Pharmacy Co., LTD., Jeonnam 58144, Korea; ij.han@koreaprime.co.kr

**Keywords:** flavor, mushroom, fresh, dried, sensory, descriptive, lexicon

## Abstract

Mushrooms are a nutritious versatile ingredient in many food products. They are low in calories and have various potential medicinal properties as well. Surprisingly, little research on their descriptive sensory properties has been conducted. The objectives of this study were to a) establish a descriptive sensory flavor lexicon for the evaluation of fresh, dried, and powdered mushrooms and 2) use that lexicon to compare a selection of different mushrooms of various species and in fresh dried and powdered forms. A lexicon for describing mushroom was developed using a consensus profile method. A highly trained, descriptive sensory panel identified, defined, and referenced 27 flavor attributes for commercially available mushroom samples prepared as “meat” and broth. Attributes could be grouped in categories such as musty (dusty/papery, earthy/humus, earthy/damp, earthy/potato, fermented, leather (new), leather (old), mold/cheesy, moldy/damp, mushroomy), and other attributes such as fishy, shell fish, woody, nutty, brown, green, cardboard, burnt/ashy, potato, umami, protein (vegetable), yeasty, bitter, salty, sweet aromatics, sour, and astringent. Samples were then tested in three replications and mean values were compared statistically. In addition, principal component analysis was used to understand the characteristics of mushrooms evaluated. Dried mushrooms showed bitter, burnt, musty/dusty, astringent, old leather, and fresh mushroom characteristics and fresh mushroom showed umami, sweet, earthy/potato, earthy/damp, yeasty, and fermented. Mushrooms were grouped and differentiated in similar ways regardless of whether they were tested as broth or “meat”. Mushroom growers, product developers, chefs and other culinary professionals, sensory scientists, researchers, the food industry, and ultimately consumers will benefit from this lexicon describing a wide variety of mushroom flavor properties.

## 1. Introduction

Mushrooms are defined as “a macro-fungus with a distinctive fruity body which can be either epigeous (above ground) or hypogenous (underground) and large enough to the seen with the naked eye and to be picked by hand” [1]. Although more than 2000 species, including some wild species, are potentially edible [2], fewer are widely available or used commercially in food. Mushrooms can be found in many recipes and have been used as food or food ingredients for millennia [3]. They are recognized as important sources of fiber, micronutrients and functional compounds [4,5,6]. Vegetarian diets often include mushrooms, which increases the diversity of essential amino acids available in the diet [6]. Many studies have shown various effects of mushrooms or mushroom extracts on potential reduction or improvement of health issues such as cancer, heart disease, diabetes [5,7,8,9,10,11,12]. They also meet the criteria cited by researchers for being described as a “natural” ingredient [13,14].

The flavor compounds of various types of mushrooms and processed mushrooms have been elucidated. In general, alcohols, ketones, aldehydes and cyclic compounds, particularly C8 (8 carbon) compounds, are major flavor components [15,16]. Compounds such as 1-octen-3-ol, (E)-2-octen-1-ol and geranyl acetone have been shown to be related to mushroom-like flavor [17]. In fact, 1-octene-3-ol has been described as intensely mushroom-like [18] at 10,000 ppm. The compounds vary in amount and type depending on the mushroom and the processing. When processed, even simple steps such as chopping or grinding release enzymes that alter the chemical structure and composition of the mushrooms [15]. For example, chopped mushrooms had higher levels of 3-octanone than homogenized samples, but the homogenized samples were higher in 1-octen-3-ol [15]. Higher levels of processing, such as drying, produce changes both in levels of C8 compounds (usually reducing those levels) and in the formation of new compounds such as Maillard reaction products [16,17,19]. Chen and others [17] found nitrogen- and sulfur-containing compounds after heating mushroom hydrolysates. Compounds such as 3-phenylfuran and 2-octylfuran (caramel-like flavor) and 2-thiophene-carboxaldehyde and 2,5-thiophenedicarboxaldehyde (meat-like flavors) were formed during their heating process.

There is far less information on the sensory flavor aspects of mushroom. Authors have identified general characteristics such as “fresh shiitake flavor” [16] and others have focused on the umami and “kokumi” taste potential of mushrooms [19,20,21,22,23,24] showing that the free amino acids and ribonucleotides in mushroom can heighten flavor characteristics. Guinard et al. [25] demonstrated that partial substitution of mushroom into meat dishes with concomitant sodium reduction did not decrease consumer acceptance. Other authors have shown that acceptance of mushroom addition to a meat patty is both a function of amount of mushroom added and culture, with higher levels being acceptable by Korean consumers and lower levels accepted by U.S. consumers [26]. Various authors have examined sensory properties of specific mushrooms added to food products e.g., [25,26,27,28,29] but many of those studies focused on consumer acceptance rather than flavor properties. Moliszewska [30] provided a review of some specific aroma notes found in a few mushroom varietals and related some of those notes to chemical compounds present in those particular mushrooms. The author noted that the aroma of mushrooms ranges from typical mushroom flavors to floral, fruity, vegetable, herb, and even fecal notes, but that work focused more on the ability of mushrooms to provide compounds useful for perfumery and fragrances rather than the notes inherent in mushrooms typically used in food. However, no research was found on the overall flavor characteristics of mushrooms commonly used in food that could help to understand the broad category of mushroom flavor from a fresh, cooked, and dried perspective. Perhaps that is because a lexicon for such sensory work is lacking. Such lexicons have been published for many products over the years including meat [31,32], fish [33,34,35], vegetables [36,37,38,39,40], fruits [41,42,43,44,45], grains and breads [46,47,48], dairy products [49,50,51,52,53,54,55,56], beverages [57,58,59,60,61], other processed foods [62,63,64,65,66,67], and types of chemical compounds [68,69,70,71]. Suwonsichon [72] stated that lexicons are “an effective communication tool and a guidance tool for new product development processes, quality control, product improvement, measuring changes during product shelf life, and breeding new plant cultivars”.

The objectives of this research were (1) to develop a lexicon of selected commonly available mushrooms used in food preparation including fresh, dried, and powdered forms, and (2) to develop a “map” of the flavor of those mushrooms.

## 2. Materials and Methods 

### 2.1. Samples

Samples of fresh and dried mushrooms and mushroom powder were obtained locally at supermarkets and specialty markets or were obtained from internet sources. The list of samples is provided in Table 1.

Fresh samples were stored for 2 days or less than 2 days after receipt and dried and powdered samples were held at room temperature for up to 2 weeks before testing. Samples were stored in their original containers whenever possible.

To test the “meat” (cap and stipe), fresh mushroom samples were cleaned by rinsing in purified water (treated by reverse osmosis and carbon filtration), gently patted with cotton toweling to remove excess moisture, and allowed to dry out at room temperature for 30 minutes before cutting into 0.5 cm thick samples and serving. Dried samples were rehydrated by covering the samples in purified water and allowing them to rehydrate for 2 hours before serving. They were then carefully tossed in the soaking water to remove any grit remaining. Dried samples were not rinsed after soaking. Powdered samples were not included in the fresh sampling.

To test the mushroom “broth”, an established amount of mushroom was added to purified boiling water (fresh: 200 g mushroom to 1 L water; dried: 30 g mushroom to 1 L water; powdered: 3 g mushroom to 300 mL water) and simmered (fresh: 1–3 min. depending on mushroom; dried: 1–15 min. depending on mushroom; powdered: 2 min.). Broth was served at 71 °C in polystyrene foam bowls placed on warmed tiles to maintain temperature.

### 2.2. Panelists

All sensory testing conducted for this project was approved by the Kansas State University Institutional Review Board on Human Subjects Research as “Exempt” (Taste Tests) under protocol #5826.

Six highly trained panelists from the Center for Sensory Analysis and Consumer Behavior, Kansas State University, Manhattan, KS comprised the descriptive panel for this study. The panelists had completed 120 h of training on a broad range of products in all aspects of descriptive sensory techniques, including attribute identification and scaling. Panelists also had more than 1000 h of testing experience in general sensory testing for a wide variety of foods, including multiple studies on products containing mushroom. Each panelist had participated in periodic revalidation and retraining during their tenure in sensory testing. Such numbers of highly trained panelists have been shown to be able to discriminate among samples better than larger panels of less trained panelists [73,74,75]. The research was conducted with approval from the Institutional Review Board for Human Subjects.

### 2.3. Sample Evaluation

Panelists used a consensus descriptive sensory method [76] used in other studies [41,46,64,77,78,79,80,81] for lexicon development. Individual panelists first evaluated samples representing the broad range of mushrooms that would be tested and made notes on the characteristics present. Then, the panel leader led a group discussion to reach agreement on the identified descriptors. As the sampling progressed, the lexicon as a whole was discussed to agree on the descriptors, define the flavor notes more precisely, suggest definitions and potential references (including foods and chemicals) that might represent the characteristics, and to ensure that multiple attributes were not used to describe the same flavor. Discussion and tasting continued until the panel developed a lexicon. Product or chemical references that helped illustrate the flavor were determined and scored on a 0–15 point scale divided into half-point increments and ranging from 0 (none) to 15 (extremely strong). This process of initial lexicon development and references took four 1.5 h sessions. Once the basic lexicon was established, testing was conducted on each mushroom sample, fresh or broth. Samples were tested individually in random order within each of the three replications using the 0–15 scale. Purified water, unsalted crackers, and carrots were provided to cleanse the palate during testing. Twelve sessions (one per day) of 1.5 h each with 8–9 samples tested per session were held for testing mushrooms and 12 sessions were held for testing broth.

### 2.4. Statistical Analysis

Analysis of variance (ANOVA) with Fisher’s protected least significant difference (LSD) was used to compare samples. Principal component analysis with mapping was conducted using the mean values for each sample to produce a 2-dimensional map of samples to make understanding of the major differences easier.

## 3. Results and Discussion

### 3.1. Lexicon

The final lexicon determined by the panel is shown in Table 2.

#### 3.1.1. Musty Attributes

There are a number of attributes that revolve around the concept of mustiness that is quite apparent in most mushrooms. That musty concept is divided into various aspects that show different types of mustiness. Many of these attributes are similar to those used in previous research on musty chemical compounds [18] and mustiness of grain samples [82,83], but are particularly prevalent in this study of mushrooms. Although each of the attributes is part of the overall mustiness exhibited in mushrooms, each is distinctly different in character. Those differences are obvious in terms such as dusty/papery and earthy/damp which clearly describe differences in a wet and dry musty character. It may be less obvious to the reader in attributes such as new leather and old leather, which are more similar in character but still describe distinctly different odor/flavors. Those are better understood by examining the differences in definitions and in differences in references used as examples of the odor. 

#### 3.1.2. Non-Musty Attributes

Other attributes associated with mushrooms such as fishy, woody, brown, umami, and salty provide breadth and depth of flavor experience for various types of mushrooms. This particular blending and heightening of flavors, which is not unique to mushrooms but is exhibited broadly in products containing mushrooms, is often known as “amplitude” in flavor literature [76,84,85].

The range of attributes present in these samples of mushrooms is interesting because it encompasses attributes common in many different types of food products. For example, some of the attributes are part of what might be “savory” or protein-based food attributes such as fishy, shellfish, protein, and umami. Forde and others [86] showed that most meat and fish products were much more savory than many vegetable or grain-based products. Some attributes were more associated with plant-based materials such as green, potato, woody, and nutty. Such attributes have been described by authors in association with vegetables, nuts, nut spreads, grains/cereals, seeds, beans/legumes, oils, beverages, as well as some animal-based foods such as cheese [36,38,65,68]. Some attributes may be more associated with processing, packaging, or shelf-life such as brown, cardboard, burnt/ashy, and yeasty notes that appear in many roasted, processed, and products including nuts, tea, coffee and meat [58,87,88,89]. Of course, those attributes, along with bitter, salty sweet aromatics, sour, and astringent can also appear in many other foods as well. This expanse of flavors shows the potential that the mushroom family has for blending with other foods and boosting flavor characteristics as well as providing additional flavors to foods and recipes where needed.

### 3.2. Intensities of Flavor in Various Mushroom Broths

First, it must be noted that because we used a universal-type sensory scale where the range of scores went from none to extremely high, most of the scores for mushroom flavor are quite low because mushroom typically is a slight, delicate flavor. That did not appear to affect our ability to find differences in this research given the small least significant differences (LSDs) from the statistical tests. However, in the future, researchers may want to use a product-specific scale that allows smaller differences to be highlighted, but this can make comparisons among studies more difficult [46].

Intensities of musty flavors for mushroom broth are shown in Table 3. The intensity comparisons for mushroom meat and broth were surprisingly similar and because the meat comparison did not contain the powdered mushroom samples, they are not included in the tabular data (those comparisons are shown in the figures).

The data show that mustiness values are generally low, but do change depending on the species and processing of the mushrooms tested. For example, drying mushrooms tends to change their character. For example, Black Trumpet mushroom was characterized as a potato-like mustiness when fresh and made into broth, but changed to a dusty/papery, earthy/humus, earthy/damp, and old leather character when dried and used to make broth. In contrast, fresh Portobello had an earthy/damp character that increased slightly when dried, but it also increased in dusty/papery and moldy/damp character. Qin et al. [90] suggested that activation of enzymes during drying produced sulfur-containing compounds that change the flavor of mushrooms. Such compounds are also found in the mustiness of onions and musty breath odor [91].

Mushrooms with the most “mushroom” character spanned both fresh (Baby Portobello, Portobello, Button) and dried (Oyster, Shiitake) mushroom categories. Interestingly, some of these are the most commonly consumed mushrooms in the world [92]. Perhaps the fact that many other mushrooms have less “mushroom” flavor could make them more popular with people who typically do not like that flavor.

It is of special note that a few of the mushrooms tested had little musty character at all. For example, in the dried category both Cloud Ear and Lobster had no musty scores as high as 1.0. For fresh, Alba Clamshell, Brown Clamshell, nor Oyster had any musty scores as high as 1.0.

Table 4 highlights the many other flavors contained in mushrooms. Some of the notes such as woody, nutty, brown, bitter, sweet aromatics, sour, and astringent are common to a number of different mushrooms, but at varying intensities. Some flavor notes such as fishy or yeasty are found at quite low levels and at mean levels that are noticeable in only a few samples such as Enoki for both fishy and yeasty. Some flavor notes appear to be unique to certain categories of mushrooms such as burnt/ashy, which appear at noticeable levels only in dried and powdered mushrooms. This may be caused by drying temperatures that are too high or too long resulting in caramelization of carbohydrates in the mushrooms, as these flavors typically are not associated with the brown, toasted notes from Maillard reactions.

The umami flavor present in the mushrooms is quite variable, which was not expected. Most publications about umami in mushroom [17,19,24,93,94] focus on specific mushrooms that contain umami-enhancing compounds such as amino acids (glutamic acid for example), 5′ nucleotides and other various compounds. This has led to a belief that all mushrooms have “umami”, which this research shows is not the case. What was not clear in those previous studies is the umami-enhancing ability of mushrooms that have not been as widely studied, such as Wood Ear and Clamshell, which seem to have little umami flavor and, thus, may have little of the “savory” enhancement that goes with that flavor. This makes those mushrooms ideal candidates for foods in which a savory character is not needed or is not desirable, but where the other characteristics of flavors are wanted.

### 3.3. Principal Components Mapping of Flavors and Mushroom Samples

**Figure 1:**
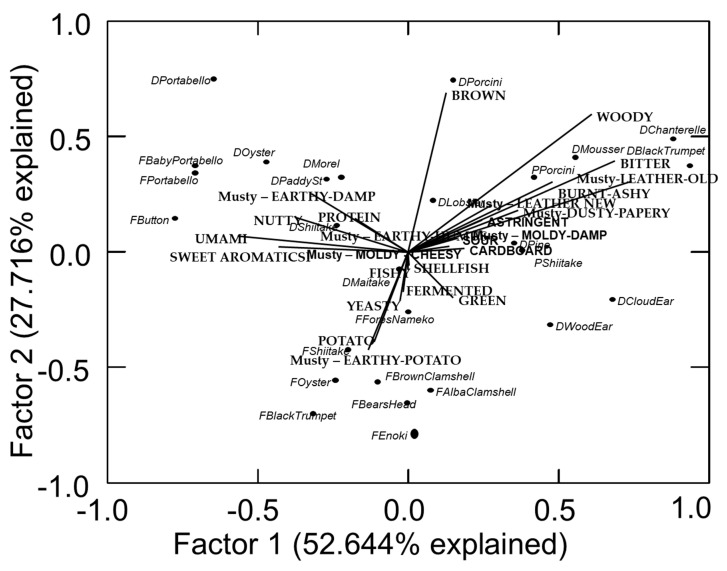
Principal component map of flavor notes and mushroom broth (fresh and reconstitued dried).

The PCA map of the flavors and mushroom broth samples is shown in Figure 1. Dimension 1 shows those attributes primarily focused on musty-earthy-damp, nutty, umami, and sweet aromatics on the left side of the figure vs the musty-dusty/papery, musty-earthy-humus, cardboardy flavor notes on the right side. Four mushroom samples, dried Portobello, fresh Portobello, fresh Baby Portobello, and fresh Button (all the same species, just different ages), group close together on the left side suggesting that they are similar in flavor. Dried Cloud Ear and dried Pine grouped closest together on the right side of the map, but did not particularly group near any specific attribute, perhaps suggesting they were more related to low levels of attributes rather than high levels of any specific set of attributes.

The second dimension shows brown and woody at the top of the map with dried Porcini, Chanterelle, and Mousseron mushrooms closest to those attributes. On the opposite lower side of the map, potato, musty-earthy-potato, yeasty, and fermented attributes are closest to a range of fresh mushrooms including Shiitake, Oyster, Black Trumpet, both Clamshells, Bears Head, and Enoki. It is critical, however, to refer back to the tabular mean values because while these maps present an easy to understand condensed version of the data, maps can be misleading in some ways [95,96]. As shown here, the yeasty attribute vector appears to fall in the same direction as various samples that have quite low levels of yeasty flavor notes. Only the Enoki scored higher than 1.0 for yeasty character.

**Figure 2:**
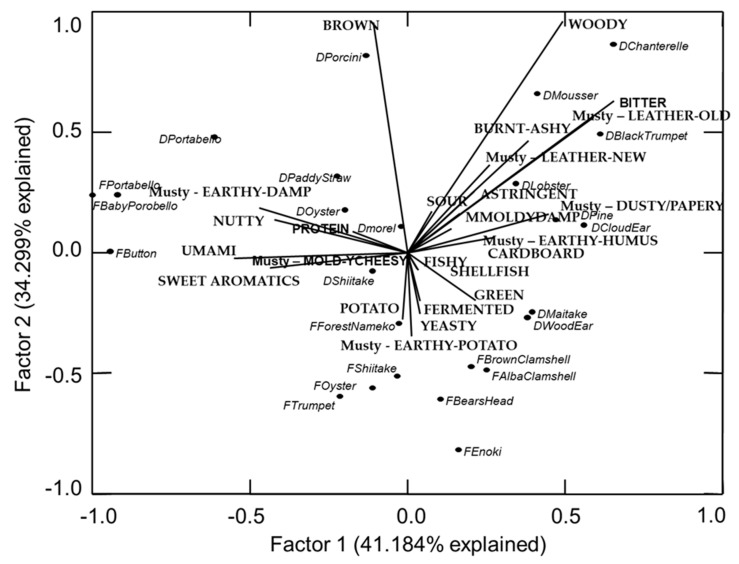
Principal component map of flavor notes and mushroom meat (fresh and reconstituted Dried).

As mentioned, the scores for the “meat” of the mushrooms visually mapped similarly to the mushroom “broth” with only a little variation (Figure 2). Almost all of the samples exist in the same quadrant they did for the broth and the flavor notes are similarly distributed in the map as well. Some small differences are noted. For example, dried Pine and dried Wood Ear have moved closer together in the “meat” map, but still fall into the same general areas as they did in the broth map. This provides another positive for developers and chefs who want to use mushrooms for flavor, but do not necessarily want to impart the texture or visuals of mushroom. Because the flavor is similar between the actual mushroom meat and “extracted” broth, either might be used to impart the flavor. Further work in culinary dishes where mushroom is used as an ingredient needs to be done to determine if this effect holds true in actual food products and not just in mushroom meat and broth.

## 4. Conclusions

A lexicon for a range of mushrooms was developed and used to describe and differentiate mushrooms. A lexicon should not be considered fixed or final and as suggested by other authors [58] serves as a point from which other researchers studying mushrooms or other products can begin using well-documented terms directly, can add to, or can adapt terms to their own work as needed. The research also showed that some beliefs, such as those related to the idea that all mushrooms have typical mushroom flavor notes and contain umami flavor, are incorrect. This research shows that mushrooms have a variety of flavor notes that can be used in culinary and product development to enhance products and provide unique flavor experiences for consumers.

## Figures and Tables

**Table 1:** Mushrooms used in the research.

Form	Common Name	Scientific Name
Fresh	Alba Clamshell	*Hypsizygus tessulatus*
	Baby Portobello	*Agaricus bisporus*
	Bears Head	*Hericium erinaceus*
	Black Trumpet	*Craterellus fallax*
	Brown Clamshell	*Hypsizygus tessulatus*
	Button	*Agaricus bisporus*
	Enoki	*Flammulina velutipes*
	Forest Nameko	*Cantharellus cibarius*
	Oyster	*Pleurotus spp.*
	Portobello	*Agaricus bisporus*
	Shiitake	*Lentinula edodes*
Dried	Black Trumpet	*Craterellus fallax*
	Chanterelle	*Cantharellus cibarius*
	Cloud Ear	*Auricularia auricular*
	Lobster	*Hypomyces lactifluorum*
	Maitake	*Grifola frondosa*
	Morel	*Morchella esculenta*
	Mousseron	*Marasmius oreades*
	Oyster	*Pleurotus spp.*
	Paddy Straw	*Volvariella volvacea*
	Pine	*Tricholoma magnivelare*
	Porcini	*Boletus edulis*
	Portobello	*Agaricus bisporus*
	Shiitake	*Lentinula edodes*
	Wood Ear	*Auricularia auricular*
Powdered	Porcini	*Boletus edulis*
	Shiitake	*Lentinula edodes*

**Table 2:** Lexicon for flavor description of mushrooms studied in this research.

Attribute	Definition	Reference ^ab^ and Intensity ^c^
**Musty**		
Dusty/Papery	Dry, musty, papery.	2,3,4-trimethoxybenzaldehyde (neat) = 4.0 (aroma)
Earthy/Humus	Musty, sweet, decaying vegetation.	1000 PPM of 2-6-Dimethylcyclohexanol. (in Propylene glycol) = 9.0 (aroma)
Earthy/Damp	Musty, damp, wet soil.	1000 PPM of Geosmin (in water) = 4.0 (aroma) Miracle-Gro Enriched Potting Mix = 11.0 (aroma)
Earthy/Potato	Musty, dry soil, potato-like.	100,000 PPM of 1,2,4-trimethoxybenzene (in Propylene glycol) = 7.0 (aroma) Baked Potato peel = 10.0 (aroma)
Fermented	Sweet, overripe, rotten, and musty.	10,000 PPM of 3-octanone(in Propylene-glycol) = 8.0 (aroma) Blackberry WONF 3 RA 654 (International Flavors and Fragrances) = 10.0 (aroma)
Leather (New)	Musty, new leather (like new shoes or purses).	1000 PPM of 2-6-Dimethylcyclohexanol (in Propylene glycol) = 4.0 (aroma)
Leather (Old)	Musty, old leather (like old book bindings).	2,3,4-trimethoxybenzaldehyde (neat) = 3.0 (aroma)
Moldy/Cheesy	Sour, musty, moldy.	10,000 PPM of 3-octanone (in Propylene glycol) = 7.0 (aroma) Joan of Arc Brie de Luxe Cheese = 10.0 (aroma)
Moldy/Damp	Musty, damp basement-like, earthy, moldy.	2,3,4-trimethoxybenzaldehyde (neat) = 3.0 (aroma)
Mushroomy	Slightly musty, earthy.	Monterey Clean N ready Baby White Pearls (Raw Button mushroom) = 9.0 (aroma)
**Non-Musty**		
Fishy	Aromatics associated with fish.	Kelp (Wando, Korea) solution = 4.0 (aroma)
Shellfish	Aromatic associated with shellfish such as clam, shrimp, oyster, and crab.	Reese’s Clam Juice (diluted) = 12.0 (aroma)
Woody	The flat, dark, dry aromatics associated with the bark of a tree.	Diamond Pecan Halves = 3.5 (flavor)
Nutty	A light, slightly sweet, brown aromatic associated with wheat germ and certain whole grains.	Diamond Pecan Halves = 9.0 (flavor)
Brown	A rich, full aromatic impression always characterized as some degree of darkness, generally associated with other attributes, i.e., toasted, nutty, sweet, etc.	Diamond Pecan Halves = 7.5 (flavor)
Green	Aromatics associated with green vegetables and newly cut vines.	Tomato vine = 7.0 (aroma)
Cardboard	Aromatics associated with cardboard that may include a stale character.	Cardboard pieces with water in covered snifter = 7.5 (aroma)
Burnt/Ashy	A charred scorched aromatics.	FMV Puffed Wheat Cereal = 7.0 (flavor)
Potato	The starchy, slightly metallic, cooked vegetable-like character associated with the meat of a baked potato.	Baked Potato = 8.0 (flavor)
Umami	Flat, salty flavor enhancers naturally occurring in some mushrooms.	0.35% Accent Salt Solution = 7.5
Protein (vegetable)	Aromatics associated with extracts or broth of mushrooms. May have a brown character.	1% Mushroom Soup Stock solution = 5.0 (flavor)
Yeasty	A sour, fermented aromatic commonly associated with yeast.	Wonder Bread Big Slice = 4.0 (flavor) Fleischmann’s Yeast = 13.5 (aroma)
Bitter	The fundamental taste sensation of which caffeine or quinine are typical	0.01% Caffeine Solution = 2.0 0.02% Caffeine Solution = 3.5 0.035% Caffeine Solution = 5.0 0.05% Caffeine Solution = 6.5 0.06% Caffeine Solution = 8.5
Salty	The fundamental taste sensation of which sodium chloride is typical.	0.15% Sodium Chloride Solution = 1.5 0.2% Sodium Chloride Solution = 2.5
Sweet Aromatics	Aromatics associated with the impression of sweet substances.	Nabisco Lorna Doone Cookie = 4.5
Sour	The fundamental taste sensation of which citric acid is typical.	0.015% Citric Acid Solution = 1.5 0.025% Citric Acid Solution = 2.5
Astringent	The complex of drying, puckering, shrinking sensations in the oral cavity.	0.05% Alum Solution = 2.5 0.1% Alum Solution = 5.0

^a^ References were served at room temperature. ^b^ It is important to note that actual reference may vary across markets and countries and are included as examples. They may need to be changed or adapted to the specific research situation. ^c^ Intensities based on a 0 = none to 15 = extremely strong scale.

**Table 3:** Mean intensity of musty flavors in selected mushroom broths (0 = none to 15 = extremely high).

Form	Product	Musty: Dusty Papery	Musty: Earthy/Humus	Musty: Earthy/Damp	Musty: Earthy/Potato	Musty: Fermen ted	Musty: Leather/New	Musty: Leather/Old	Musty: Moldy/Cheesy	Musty: Moldy/Damp	Musty: Mushroomy
Dried	Black Trumpet	1.9	0.6	0.8	0.0	0.1	1.0	2.6	0.1	1.4	0.2
Dried	Chanterelle	1.4	0.1	0.4	0.0	0.0	1.8	2.3	0.0	0.3	0.1
Dried	Cloud Ear	0.9	0.9	0.3	0.0	0.0	0.1	2.6	0.0	1.3	0.1
Dried	Lobster	0.9	0.1	0.8	0.1	0.1	1.0	0.7	0.0	1.0	1.5
Dried	Maitake	2.0	0.1	1.4	0.4	0.2	0.1	0.3	0.1	0.7	1.8
Dried	Morel	1.2	0.2	1.8	0.1	0.0	0.3	0.3	0.1	0.8	2.3
Dried	Mousseron	1.6	0.2	0.6	0.1	0.0	0.6	2.0	0.0	0.7	0.9
Dried	Oyster	1.0	0.1	1.5	0.1	0.0	0.2	0.4	0.0	0.3	3.6
Dried	Paddy Straw	1.4	0.1	0.9	0.2	0.1	0.9	0.1	0.4	0.6	2.3
Dried	Pine	1.6	0.5	1.0	0.1	0.1	0.8	1.2	0.3	1.2	1.0
Dried	Porcini	1.2	0.2	1.8	0.0	0.1	0.8	0.9	0.0	0.5	1.6
Dried	Portabella	0.8	0.1	1.9	0.1	0.0	0.0	0.1	0.0	0.5	3.9
Dried	Shiitake	1.2	0.0	1.5	0.4	0.0	0.1	0.2	0.1	0.2	2.1
Dried	Wood Ear	1.5	0.1	0.4	0.0	0.0	0.6	1.0	0.0	0.3	0.3
Fresh	Alba Clamshell	0.7	0.0	0.8	0.9	0.1	0.1	0.0	0.0	0.6	1.2
Fresh	Baby Portabella	0.4	0.0	1.8	0.2	0.0	0.0	0.1	0.0	0.7	3.7
Fresh	Bears Head	0.5	0.1	0.6	0.8	1.3	0.0	0.0	0.0	0.4	0.9
Fresh	Brown Clamshell	0.7	0.0	1.0	0.8	0.1	0.0	0.1	0.0	0.2	1.4
Fresh	Button	0.0	0.0	1.6	0.1	0.0	0.0	0.0	0.0	0.3	3.6
Fresh	Enoki	0.6	0.0	0.7	1.5	0.9	0.0	0.0	0.0	0.5	0.9
Fresh	Forest Nameko	1.1	0.4	0.8	0.6	0.2	0.4	0.2	0.0	0.2	1.1
Fresh	Oyster	0.8	0.0	0.8	0.8	0.0	0.0	0.0	0.0	0.0	1.3
Fresh	Portabello	0.1	0.0	1.5	0.1	0.1	0.0	0.0	0.0	0.1	3.6
Fresh	Shiitake	0.4	0.0	0.7	1.0	0.7	0.0	0.0	0.5	0.4	1.5
Fresh	Black Trumpet	0.2	0.0	0.5	1.6	0.1	0.0	0.0	0.0	0.1	1.5
Powder	Porcini	1.4	0.0	0.6	0.0	0.0	0.3	1.6	0.0	0.2	1.1
Powder	Shiitake	1.4	0.4	1.1	0.1	0.0	0.3	1.3	0.0	0.6	0.8
Least Significant Difference *		0.3	0.2	0.3	0.2	0.1	0.3	0.3	0.1	0.2	0.3

* Any difference in means of this size or larger is significantly different.

**Table 4:** Mean intensity of other flavors in selected mushroom broths (0 = none to 15 = extremely high).

Form	Product	Fishy	Woody	Nutty	Brown	Green	Cardboard	Burnt Ashy	Potato	Umami	Protein	Yeasty	Bitter	Sweet Aromatics	Sour	Astringent
Dried	Black Trumpet	0.0	2.8	0.1	2.3	0.6	0.8	2.2	0.0	0.1	0.0	0.1	5.4	0.4	1.3	1.9
Dried	Chanterelle	0.0	3.4	0.3	2.9	0.0	0.8	1.6	0.0	0.0	0.1	0.0	5.2	0.1	0.9	2.1
Dried	Cloud Ear	0.0	1.7	0.0	1.6	1.5	0.1	0.9	0.1	0.1	0.0	0.0	3.7	0.6	1.9	1.6
Dried	Lobster	0.0	1.8	0.9	2.0	0.1	0.9	1.3	0.0	0.9	0.4	0.1	3.3	1.2	0.9	1.4
Dried	Maitake	0.0	0.9	0.4	1.4	0.4	0.8	0.7	0.3	0.9	0.1	0.2	3.2	1.1	0.9	1.4
Dried	Morel	0.0	1.4	0.9	1.9	0.0	0.3	0.8	0.2	1.3	0.4	0.0	3.4	1.4	0.9	1.4
Dried	Mousseron	0.0	2.7	0.4	2.6	0.1	0.7	1.7	0.0	0.4	0.2	0.1	4.4	0.7	1.0	1.6
Dried	Oyster	0.0	0.9	1.1	1.7	0.1	0.2	0.3	0.2	1.5	0.6	0.0	3.6	1.3	0.9	1.3
Dried	Paddy Straw	0.0	1.5	1.4	2.1	0.0	0.2	0.4	0.3	1.6	0.3	0.2	3.3	1.9	0.8	1.2
Dried	Pine	0.0	1.4	0.4	1.4	0.5	0.6	1.4	0.1	0.7	0.0	0.2	4.1	1.1	1.2	1.5
Dried	Porcini	0.0	2.6	0.9	3.4	0.1	0.1	1.0	0.0	1.0	0.7	0.1	4.7	0.9	1.2	1.9
Dried	Portabella	0.0	1.4	1.5	2.8	0.0	0.1	0.8	0.1	1.7	0.9	0.0	3.2	1.8	1.0	1.3
Dried	Shiitake	0.0	1.2	0.8	1.7	0.1	0.6	0.4	0.3	1.5	0.7	0.1	3.4	1.4	0.8	1.4
Dried	Wood Ear	0.0	1.6	0.1	1.1	0.2	0.9	1.0	0.0	0.0	0.0	0.0	3.0	0.1	0.8	1.5
Fresh	Alba Clamshell	0.4	0.6	0.4	0.6	1.5	0.3	0.3	0.8	0.8	0.1	0.2	3.8	0.7	1.0	1.4
Fresh	Baby Portabella	0.0	1.0	1.2	1.9	0.0	0.1	0.2	0.2	1.9	0.8	0.1	2.3	1.7	0.9	1.1
Fresh	Bears Head	0.0	0.7	0.4	0.9	0.5	0.2	0.1	1.0	1.1	0.3	1.2	3.2	1.1	0.7	1.4
Fresh	Brown Clamshell	0.3	0.2	0.6	0.8	0.5	0.3	0.1	0.9	0.9	0.1	0.2	3.1	1.0	0.7	1.4
Fresh	Button	0.0	0.2	1.1	1.6	0.1	0.0	0.0	0.1	2.0	0.4	0.1	2.1	1.5	0.4	0.8
Fresh	Enoki	0.6	0.1	0.3	0.3	0.8	0.3	0.2	1.8	1.0	0.1	1.2	3.1	1.3	0.9	1.2
Fresh	Forest Nameko	0.0	1.3	0.5	1.6	0.1	0.4	0.3	0.5	0.9	0.2	0.5	2.8	0.9	0.7	0.9
Fresh	Oyster	0.0	0.2	0.9	0.9	0.1	0.4	0.1	1.1	1.2	0.2	0.1	2.3	1.3	0.5	0.9
Fresh	Portabello	0.0	0.8	1.3	2.1	0.0	0.0	0.2	0.2	1.9	0.6	0.2	2.6	1.6	0.6	1.1
Fresh	Shiitake	0.0	0.6	0.9	1.1	0.1	0.1	0.6	0.6	1.3	0.2	0.5	2.7	1.2	0.8	1.0
Fresh	Black Trumpet	0.0	0.3	1.1	1.1	0.1	0.1	0.1	1.7	1.1	0.1	0.4	2.0	1.7	0.6	0.8
Powder	Porcini	0.0	2.3	0.2	2.4	0.2	0.3	1.6	0.1	0.6	0.2	0.0	4.5	0.6	1.0	1.6
Powder	Shiitake	0.0	1.4	0.2	1.6	0.1	0.6	1.4	0.1	0.5	0.1	0.0	3.9	0.5	1.0	1.4
Least Significant Difference (LSD) *		0.1	0.2	0.2	0.3	0.1	0.2	0.2	0.2	0.2	0.2	0.1	0.3	0.2	0.2	0.2

* LSD indicates any difference in scores of this size or larger is significantly different.
